# Metrical analysis of disc-condyle relation with different splint treatment positions in patients with TMJ disc displacement

**DOI:** 10.1590/1678-7757-2016-0471

**Published:** 2017

**Authors:** Mu-Qing Liu, Jie Lei, Jian-Hui Han, Adrian U-Jin Yap, Kai-Yuan Fu

**Affiliations:** 1 Peking University School & Hospital of Stomatology, Center for TMD & Orofacial Pain and Department of Oral & Maxillofacial Radiology, Beijing, China; 2 National Engineering Laboratory for Digital and Material Technology of Stomatology, Beijing, China; 3 Beijing Key Laboratory of Digital Stomatology, Beijing, China; 4 Ng Teng Fong General Hospital, Department of Dentistry, Jurong Health Services, Singapore, Singapore; 5 SIM University, School of Science and Technology, Singapore, Singapore; 6 National University of Singapore, Faculty of Dentistry, Singapore, Singapore

**Keywords:** Temporomandibular joint, Mandibular condyle, Temporomandibular joint disc, Magnetic resonance imaging

## Abstract

**Objective:**

To evaluate the effect of bite positions characterizing different splint treatments (anterior repositioning and stabilization splints) on the disc-condyle relation in patients with TMJ disc displacement with reduction (DDwR), using magnetic resonance imaging (MRI).

**Material and Methods:**

37 patients, with a mean age of 18.8±4.3 years (7 male and 30 females) and diagnosed with DDwR based on the RDC/TMD, were recruited. MRI metrical analysis of the spatial changes of the disc/condyle, as well as their relationships, was done in three positions: maximum intercuspation (Position 1), anterior repositioning splint position (Position 2), and stabilization splint position (Position 3). Disc/condyle coordinate measurements and disc condyle angles were determined and compared.

**Results:**

In Position 1, the average disc-condyle angle was 53.4° in the 60 joints with DDwR, while it was −13.3° with Position 2 and 30.1° with Position 3. The frequency of successful "disc recapture" with Position 2 was significantly higher (58/60, 96.7%) than Position 3 (20/60, 33.3%). In Positions 2 and 3, the condyle moved forward and downward while the disc moved backward. The movements were, however, more remarkable with Position 2.

**Conclusions:**

Anterior repositioning of the mandible improves the spatial relationship between the disc and condyle in patients with DDwR. In addition to anterior and inferior movement of the condyle, transitory posterior movement of the disc also occurred.

## Introduction

Temporomandibular joint (TMJ) disc displacement is the most common type of TMJ arthropathy and involves an abnormal relationship or misalignment of the articular disc relative to the condyle. The usual direction for displacement of the disc is anteriorly or anterior-medially^29^. In spite of their apparent efficacy and widespread use for treating TMD, the precise mechanisms of action of oral splints remain controversial^10^. Hypotheses proposed include repositioning of condyle and/or articular disc, reduction in masticatory muscle activity, modification of patient's parafunctional behaviours, and changes in patient's occlusion^6^. Two common types of oral splints used in clinical practice are the stabilization and anterior repositioning splints.

Anterior repositioning splints (ARS) have been shown to be effective for the management of disc-condyle disorders^14^ ^,^ ^18^ ^,^ ^26^. Also known as anterior positioning appliances or mandibular orthopedic repositioning appliances, they serve to direct the mandibular condyle anteriorly in the glenoid fossa (i.e., protrusive mandibular position). The purpose of ARS treatment is not to alter the condylar position permanently, but to change it during the treatment to help the adaption of the retrodiscal tissues^24^. Based on clinical and MRI assessments, approximately 70% of reducing displaced discs was captured with the use of ARS^18^. ARS could also alter mechanical stresses on the TMJ arising from the immediate physiologic improvement in the disc-condyle relationship^4^ and has been shown to facilitate regenerative remodeling of the TMJ^22^. Although the recaptured discs can occasionally move backward with successful ARS therapy, the amount of disc movement was found to be negligible^19^. The improved condyle-disc relationship with ARS was thought to be achieved primarily by the anteroinferior movement of the condyle.

Stabilization splints (SS) cover all the maxillary and mandibular teeth and are used to treat both joint and masticatory muscle disorders^2^ ^,^ ^17^. In contrast to ARS, SS are permissive appliances (allows for teeth to glide unimpeded over the biting surface) and do not protrude the mandible. They serve to provide a temporary and removable ideal occlusion at increased vertical dimension and centric relation. The use of SS increases TMJ space^12^ and allows for antero-inferior movement of the condyles^7^ ^,^ ^11^ ^,^ ^16^. SS are also used to manage disc-condyle disorders^3^. They are effective in eliminating the signs/symptoms of TMD, except TMJ clicking^28^. When compared to ARS for the treatment of TMJ DDwR, reduction in dysfunction and TMJ symptoms were found to be lower with SS therapy^5^ ^,^ ^25^ ^,^ ^27^.

The mechanism of action of both ARS and SS remains largely hypothetical. The two oral splints with their variance in bite and mandibular positioning can produce different degrees of disc and condyle positional changes, which in turn can affect treatment outcome. Most previous MRI studies were conducted on a single splint design with the between-subject method. Thus far, few studies have compared the two splint designs using a within-subject approach (every single participant is subjected to every single splint design) and at standardized vertical dimension. This study aimed to evaluate the effects of bite positions characterizing ARS and SS therapy (with and without anterior movement) on disc/condyle locations and disc-condyle relations in patients with TMJ DDwR, using MRI metrical analysis.

## Material and Methods

### Patients

A total of 37 patients, with a mean age of 18.8±4.3 years (ranging from 12 to 30 years, 7 male and 30 female) and with complaints of TMJ clicking and/or intermittent locking, were recruited. All patients had permanent dentition, free of TMD-related pain, and 16 were younger than 18 years of age. To lessen the effect of confounding variables including marked skeletal/ occlusal discrepancies and systematic diseases, exclusion criteria were as follows: Presence of (1) crossbites and open bites; (2) deep overbites (vertical overlap of the maxillary central incisors >1/2 of the mandibular central incisors); (3) large overjets (>5 mm); (4) rheumatic or degenerative joint diseases. The study was approved by the Biomedical Institutional Review Board. Written inform consent was obtained from all participating subjects.

Fifty-one (out of 74) joints of the 37 patients were clinically diagnosed with DDwR using the RDC/TMD^9^. Bilateral DDwR was observed in 14 patients. Upon MRI examination, 9 of the 23 clinically asymptomatic joints were also diagnosed with DDwR, based on the criteria defined by Ahmad, et al.^1^ (2009). The 51 joints with clinical diagnosis of DDwR were all confirmed by MRI examination. Thus, a total of 60 joints with DDwR were included in this study. 4 asymptomatic joints were diagnosed with DDwoR and 10 joints were found to be normal with both clinical and MRI assessment.

### Determination of bite and mandibular positions

**Figure 1:**
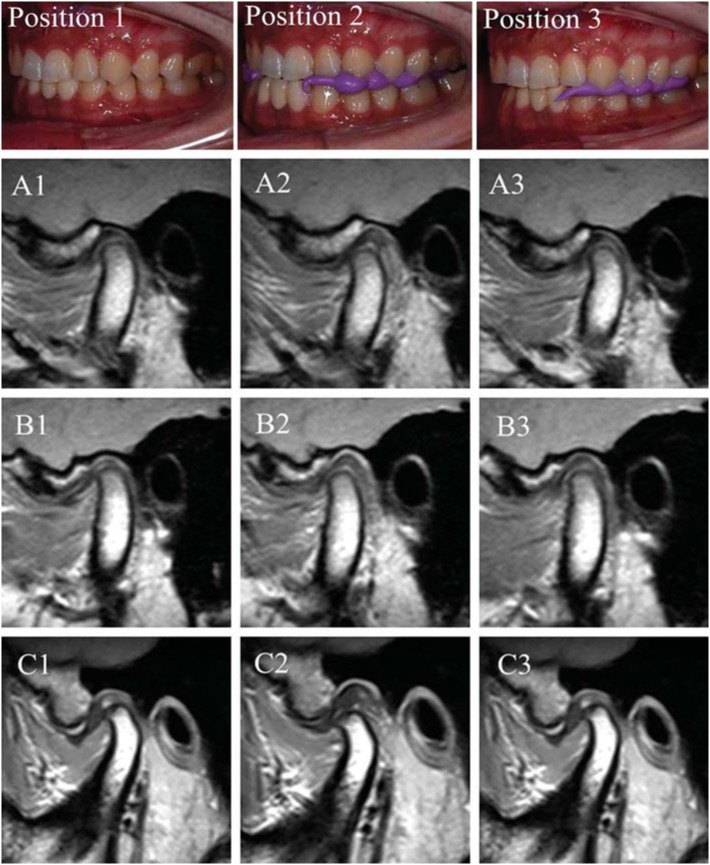
Representative MRIs of three joints in Position 1 (ICP – column 1), Position 2 (ARS – column 2), and Position 3 (SS – column 3). Joint A was normal while Joints B and C were diagnosed with DDwR

The condyle and disc locations were assessed in three bite positions: Position 1: maximal intercuspation (MICP); Position 2: characterizing ARS position; Position 3: characterizing SS position (Figure 1). For Position 1, subjects were asked to bite their back teeth completely together. The distance between the gingival margins of the left upper and lower central incisors (D1) and the overjet of the left upper central incisor were recorded using a caliper (Guanglu SF2000, Guangxi, China). For Position 2, subjects were asked to open their mouths fully beyond the clicking point and instructed to close in a protruded position with the incisors in an edge-to-edge relation. The mandibular position was registered using a silicone bite registration material (Occlufast Rock, Zhermack, Rovigo, Italy). The distance between the gingival margins of the left upper and lower central incisors (D2) was determined. For Position 3, subjects were asked to open fully beyond the clicking point and guided into the most retruded/rearmost mandibular locus. This was repeated several times till a reproducible "centric" relation position was achieved at the distance D2 without protrusion and registered. The distance between the gingival margins of the left upper and lower central incisors (D3) and the overjet of the left upper central incisor were again recorded. All bite registrations and mandibular measurements were made by a single investigator.

### Magnetic Resonance Imaging

MRI was performed with a 1.5-Tesla MR scanner (NOVUS, Siemens, Munich, Germany) with TMJ surface coils. Subjects were placed supine with their heads positioned with the Frankfurt plane perpendicular to the floor. The center beam was then lined up with the sagittal plane. All joints were scanned in the three mandibular positions in single visit using a factorial design order. For Positions 2 and 3, subjects were directed to open their mouths fully beyond the clicking point and gently close/bite into the prepared bite registrations. An initial low-resolution T1-weighted (TR 300 ms; TE 10 ms) axial localizing scan was followed by Proton-weighted (TR 1760 ms, TE 15 ms) oblique sagittal scan acquired vertical to the long axis of each condyle. The field of view was 12×12 cm and matrix size was 512×512. Slice thickness and inter-slice spacing were set at 2 mm and 1 mm, respectively.

### Metrical and statistical analysis

**Figure 2:**
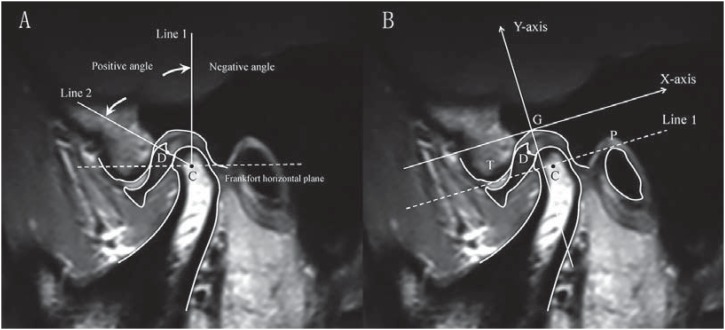
A: Drace's measurement for disc-condyle angle. C point estimated the center of the condylar head. Line 1 was drawn from C point perpendicular to the Frankfort horizontal plane. Line 2 was drawn through C point to the midpoint of the posterior margin of the posterior band of the disc (D point). The angle between line 1 and line 2 was defined as the disc-condyle angle. B: Coordinate measurement for disk and condyle position. A tangent from the lowest part of the articular tubercle (T) to the highest edge of the porus acusticus externus (P) was drawn (Line 1). The X-axis was drawn through the highest point of glenoid fossa (G), parallel to Line 1. The Y-axis was drawn from G point perpendicular to the X-axis. G point was taken as the origin of coordinates

The images were analyzed using image analysis software (Volview 3.4, Kitware, New York). The slice through the center of the horizontal long axis of the condyle was selected for evaluation (Figure 1). The disc-condyle angle was determined according to the method described by Drace and Enzmann^8^ (1990) (Figure 2A). The normal range for disc-condyle angle is between −15° to 15°^1^. Joints with disc-condyle angles greater than 15° are considered to have anterior disc displacement.

X-Y coordinate measurements were used to determine disc and condyle positions (Figure 2B). The condyle and disc positions were expressed as C and D point coordinates (x, y), respectively. The MRI data were evaluated by a trained radiologist who was blinded to clinical information. Intra-class correlation coefficients (ICC) were used to determine the intra-and inter-observer reliability. A mean intra-observer ICC of >0.900 was established for all variables (the radiologist made all measurements twice with a one-week interval). Inter-observer ICC ranged from 0.868 to 1 for the different variables. The latter was determined with the assistance of another independent radiologist who was also blinded to patients’ clinical data.

Disc and condyle X-Y coordinates and disc-condyle angles for Positions 1, 2, and 3 were compared. SPSS version 20 (SPSS IBM, Chicago, USA) was used for statistical analysis. Data was subjected to one-way ANOVA (LSD) and t-test at significance level *P values* <0.05.

## Results

### Mandibular position

The average vertical distance between the gingival margins of left upper and lower central incisors was 14.2±2.0 mm in Position 1 and 16.9±2.0 mm in Positions 2 and 3. The average overjet of the left upper central incisor was 3.1±1.1 mm in Position 1 and 3.0±1.2 mm in Position 3. Position 2 thus postured the mandible downward and forward, while Position 3 moved the mandible only downward with reference to Position 1.

### Disc-condyle angle

Disc-condyle angle in normal and DDwR joints for Positions 1 to 3 are shown in Table 1. In normal joints (n=10), no significant difference in disc-condyle angle was observed between the three positions (*P*>0.05). Disc-condyle angle was within the normal range (–15° ~+15°). In joints with DDwR (n=60), mean disc-condyle angle was reduced from 53.4° in Position 1 to −10.5° in Position 2 and 30.1° in Position 3. The percentage of displaced disc reduction or disc "recapture" (post-treatment angle between −15° and +15°) in DDwR joints was 96.7% (58/60 joints) for Position 2 and 33.3% (20/60 joints) for Position 3 (*P*<0.001). The average disc-condyle angle of DDwoR joints (n=4) in Positions 1, 2, and 3 were 82.1°, 65.5°, and 70.1°, respectively. No significant difference in disc-condyle angle was observed between the three positions (*P*>0.05).

**Table 1:** Disc-condyle angles for the three positions in normal and DDwR joints (°, mean±SD)

Positions	Normal joints(n=10)	DDwR joints(n=60)
Position 1	–1.1±10.8^a^	53.4±16.7^a^
Position 2	–11.7±12.0	–10.5±17.1^A^
Position 3	–2.7±15.5^a^	30.1±26.9^a,A^

Lowercase letters in the same row indicate significant difference between two groups (p<0.001)

Uppercase letters in the same column indicate significant difference between two positions (p<0.01)

### Coordinate measurements of condyle and disc

C points representing condylar positions in X- and Y-axis are shown in Table 2. C point movements were indicated by the numerical difference of coordinate values between two points. In normal joints, the condyle moved 2.22 mm forward and 1.49 downward from Position 1 to Position 2, and shifted 0.7 mm forward and 0.06 mm downward from Position 1 to 3. In joints with DDwR, the condyle moved 3.28 mm forward and 2.6 mm downward from Position 1 to 2, and shifted 0.97 mm forward and 0.68 mm downward from Position 1 to Position 3. Statistical analysis indicated a significantly greater forward and downward movement of the condyle with ARS position when compared to the SS position.

**Table 2:** Condyle and disc coordinates in normal and DDwR joints for Position 1, Position 2, and Position 3

Coordinates		Condyle		Disc	
		Normal joints (mm, mean±SD)	DDwR joints (mm, mean±SD)	Normal joints (mm, mean±SD)	DDwR joints (mm, mean±SD)
X coordinate	Position 1	0.11±1.40^a,A^	1.21±1.19^a,A^	1.21±0.61^b,A^	–2.02±1.50^b,A^
	Position 2	–2.11±2.14^A,B^	–2.07±1.53^A^	0.28±1.07^A^	0.21±1.67^A^
	Position 3	–0.59±1.27^B^	0.24±1.32^A^	0.71±0.77^a^	–1.27±1.92^a,A^
Y coordinate	Position 1	–7.02±0.80^a,C^	–6.36±0.94^a,B^	–1.70±0.43^b,B^	–2.81±1.05^b^
	Position 2	–8.51±0.82^C,D^	–8.96±1.10^B^	–2.34±0.41^a,B,C^	–2.96±0.81^a^
	Position 3	–7.08±0.87^D^	–7.04±1.11^B^	–1.66±0.42^a,C^	–2.76±0.92^a^

Lowercase letters in the same row indicate significant difference between two groups (p<0.05)

Uppercase letters in the same column indicate significant difference between two positions (p<0.05)

D points (posterior margin of the posterior band of disc) representing disc positions in the X- and Y-axis are also presented in Table 2. In normal joints, the D point was located 1.21 mm behind and 1.70 mm below the G point (the highest point of glenoid fossa). The disc moved 0.93 mm forward and 0.64 mm downward from Position 1 to Position 2, while the condyle moved forward and downward. The disc did not move significantly from Position 1 to Position 3 in normal joints. In DDwR joints, the disc was located 2.02 mm anterior and 2.81 mm below the G point in Position 1, indicating it was significantly displaced anteriorly and inferiorly when compared to normal joints (Table 2). In contrast to normal joints, the disc moved 2.23mm backward from Positions 1 to 2 in DDwR joints. Disc movement from Position 1 to 3 was, however, only 0.75 mm backward. The coordinate values of D point in both X- and Y-axis for Position 2 in DDwR joints were similar to normal joints, indicating that the disc was fully reduced in the protrusive position. For all the measurements, no significant difference was detected between adolescent and adult patients.

## Discussion

In this study, we determined the spatial changes in disc and condyle positions and the disc-condyle relation with mandibular positions of ARS and SS therapy in adolescents and adults. Metrical analysis was done with MRI, as it is a non-invasive diagnostic method that enables both qualitative and quantitative evaluation of the structures within the joint, including the TMJ disc. It is also generally painless and does not involve the use of ionizing radiation. As splint thickness can affect disc and condyle positions, a similar vertical dimension was maintained for both ARS and SS positions in this study. The use of mandibular positions mimicking ARS and SS instead of actual appliances negated the confounding effects of technical and clinical discrepancies associated with splint fabrication, adjustment, and use. The measurement method of disc-condyle angle and positions of condyle/disc was reliable and reproducible.

In the maximal intercuspation (Position 1), the disc in DDwR joints was displaced anteriorly and inferiorly, while the condyle was positioned backward and upward, in relation to normal joints. This corroborated the findings of earlier studies^13^ ^,^ ^20^. The condyle moved forward and downward in both ARS and SS treatment positions (Positions 2 and 3, respectively), but movement was more significant with ARS. The mechanism of action of ARS was previously thought to involve the "recapturing" of the discs, since the condyles are moved downward and forward. It was believed that the disc-condyle complex could be gradually walked back into the fossa by adjusting the biting surface of ARS^23^. Kurita, et al.^19^ (1998) found that approximately 60% of the "recaptured" disc moved posteriorly, but the amount of movement was minor. In our study, disc movement was noticeably large (2.23 mm posteriorly) in joints with DDwR for the ARS position. The D point (posterior band of the disc) actually shifted back to the G point (highest point of the glenoid fossa), indicating complete reduction of the displaced disc. In contrast, the displaced disc only moved back 0.75 mm for the SS position. The reduction of the displaced disc with ARS might be one of the key factors to the adaption and repair of the retrodiscal tissues. The elimination of joint clicking is commonly used to help determine the appropriate mandibular position for ARS^19^ ^,^ ^26^. In this study, the antero-inferior movement of the mandible for elimination of joint clicking is usually less than the protruded position with the incisors in an edge-to-edge relation. There may be a link between reduction of the displaced disc and the forward movement of the condyle. The stability of the reduced disc position, however, depends on maintaining the condyles in the forward and downward position, necessitating the full-time use of ARS over a period of time. Upon stopping ARS use, reduced discs may once again get displaced, as the condyle moves posteriorly.

A proper disc-condyle-fossa relationship is thought to be important for normal TMJ function, alleviating joint pain, preventing degenerative joint changes, and promoting mandible growth in adolescents^15^ ^,^ ^21^. Although some joints with DDwR achieved normal disc-condyle relationship in the SS position (increased vertical without mandibular anterior positioning), the percentage of disc reduction was significantly lower than with ARS (33% as opposed to 96.7% with ARS). Use of ARS achieved an immediate physiologic disc-condyle-fossa relationship and increased the prospect of disc reduction. This explains in part their superior effectiveness in decreasing pain and dysfunction in patients with DDwR when compared to SS^5^ ^,^ ^25^ ^,^ ^27^.

As with all studies, the current research has some limitations. Since actual oral splints were not fabricated, the influence of splint material stiffness on disc and condyle positions/relationships could not be ascertained. The long term effects of ARS and SS on disc-condyle-fossa relationships were also not determined. Patients with significant skeletal discrepancies, malocclusion, and rheumatic or degenerative joint diseases were excluded, which may also affect disc "recapture" in joints with DDwR.

## Conclusions

In summary, anterior repositioning of the mandible improved the spatial relationship between the disc and condyle, increasing the prospect of disc reduction in patients with DDwR. In addition to anterior and inferior movement of the condyle, transitory posterior movement of the disc also occurs with the anterior mandibular repositioning.
